# Ferroptosis: a new mechanism of traditional Chinese medicine for treating hematologic malignancies

**DOI:** 10.3389/fonc.2024.1469178

**Published:** 2024-09-23

**Authors:** Xinyue Gou, Xudong Tang, Chi Liu, Zhuo Chen

**Affiliations:** ^1^ Graduate School, China Academy of Chinese Medical Sciences, Beijing, China; ^2^ Xiyuan Hospital, China Academy of Chinese Medical Sciences, Beijing, China

**Keywords:** ferroptosis, leukemia, myelodysplastic syndromes, Chinese medicine, hematologic malignancies

## Abstract

Ferroptosis is a recently identified form of cell death characterized by lipid peroxidation and elevated iron levels. It is closely associated with hematologic malignancies, including leukemia, multiple myeloma (MM), and myelodysplastic syndromes (MDS). Research indicates that ferroptosis could represent a novel therapeutic target for these hematologic malignancies. Furthermore, traditional Chinese medicine (TCM) has been shown to modulate hematologic malignancies through the ferroptosis pathway. This paper aims to elucidate the mechanisms underlying ferroptosis and summarize the current research advancements regarding ferroptosis in hematologic malignancies, as well as the role of traditional Chinese medicine in the prevention and treatment of ferroptosis, with the goal of enhancing treatment efficacy.

## 1 Introduction

Typical forms of cell death include necrosis, apoptosis, and autophagy, each playing a critical role in maintaining body homeostasis. In recent years, ferroptosis has garnered significant attention as an emerging form of cell death. Ferroptosis is characterized as an iron-dependent, non-apoptotic form of cell death, marked by the accumulation of intracellular reactive oxygen species (1). The pathogenesis of hematological disorders is complex and encompasses a variety of disease types, with the progression of these diseases posing a significant threat to public health. Therefore, it is imperative to find effective treatments. Studies have demonstrated that ferroptosis can profoundly impact hematological function, impairing red blood cell production, inducing anemia, provoking platelet activation, and potentially leading to thromboembolism. Conversely, ferroptosis presents new potential for treating hemorrhagic cell-related diseases (2), offering improvements in conditions such as acute myeloid leukemia and T-cell leukemia. Currently, both domestic and international researches primarily investigate the effects of ferroptosis regulation on hematological diseases through the lenses of molecular mechanisms, targeted regulation, and the active ingredients of traditional Chinese medicine. This article aims to elucidate the regulatory mechanisms related to ferroptosis, summarize the latest advancements in ferroptosis-related mechanisms within hematological research, and integrate traditional Chinese medicine interventions to provide a reference for the treatment of hematological disorders.

## 2 Overview of ferroptosis

Ferroptosis was initially introduced in 2012 by Dixon et al. (1) as a form of cell death induced by the small molecule Erastin, which inhibits cystine uptake. This inhibition leads to the depletion of glutathione (GSH) and the inactivation of the phospholipid peroxidase glutathione peroxidase 4 (GPX4). Morphologically, ferroptosis does not exhibit the characteristic changes associated with apoptosis, such as cell shrinkage, chromatin condensation, and the formation of apoptotic bodies (3). Instead, it displays typical necrotic morphology, characterized by deformed mitochondria, reduced cristae, increased membrane density, and rupture of the outer membrane (4). Additionally, ferroptosis is resistant to inhibitors of apoptosis, cellular necrosis, and cellular autophagy; however, it can be inhibited by iron chelators and antioxidants. This indicates that ferroptosis is iron-dependent and is characterized by an increase in lipid reactive oxygen species (ROS). Excess iron, resulting from abnormal iron metabolism or an imbalance between the two major redox systems (lipid peroxidation and amino acids), is a primary contributor to ROS production (5). Currently, ferroptosis has been shown to effectively prevent the occurrence of tumor cells and plays a vital role in cancer treatment (6).

## 3 Mechanisms of ferroptosis

### 3.1 Iron metabolism

Disturbances in iron metabolism can lead to metabolic dysfunctions in various organs and contribute to disease. In the human body, iron primarily exists in the ferrous (Fe2+) and ferric (Fe3+) states (7). Under physiological conditions, transferrin binds to trivalent ferric ions, facilitating their entry into cells via transferrin receptors. Once inside, the intracellular metalloreductase STEAP3 reduces Fe3+ to Fe2+, allowing the release of Fe2+ into the dynamic iron pool of the cytoplasm through the divalent metal transporter protein 1. This process is integral to a wide range of subsequent physiological and biochemical reactions (8). When excess ferrous ions accumulate within the cell, an unstable iron pool is formed; the free divalent iron in the pool participates in the Fenton reaction, which generates ROS substances represented by hydroxyl radicals; The accumulation of ROS leads to the peroxidation of membrane lipids, resulting in a loss of cellular function and ultimately cell ferroptosis (9).

### 3.2 The metabolism of lipids

The massive accumulation of lipid peroxides is a hallmark of iron death, with lipid metabolic pathways playing a crucial role in this process. The peroxidation of membrane phospholipids produces phospholipid hydroperoxides (PLOOH), which are further decomposed to yield 4-hydroxynonenal, resulting in membrane instability and ultimately leading to cellular iron death. Polyunsaturated fatty acids are catalyzed by long-chain acyl-CoA synthetase 4 (ACSL4) to generate PUFA-CoA, which is then esterified by lysophospholipid acyltransferase 3 (LPCAT3) and incorporated into membrane phospholipids, forming the iron-death lipid peroxidation substrate PUFA-PL (10). Additionally, lipoxygenases (LOXs), which are non-heme iron-dependent dioxygenases, can directly oxidize biofilm PUFA to generate PLOOH (11). An increase in the expression and catalytic activity of both ACSL4 and LOXs, as well as the Fenton reaction, enhances the accumulation of lipid peroxides, ultimately leading to iron death (12).

### 3.3 System Xc- and GPX4

**Figure 1:**
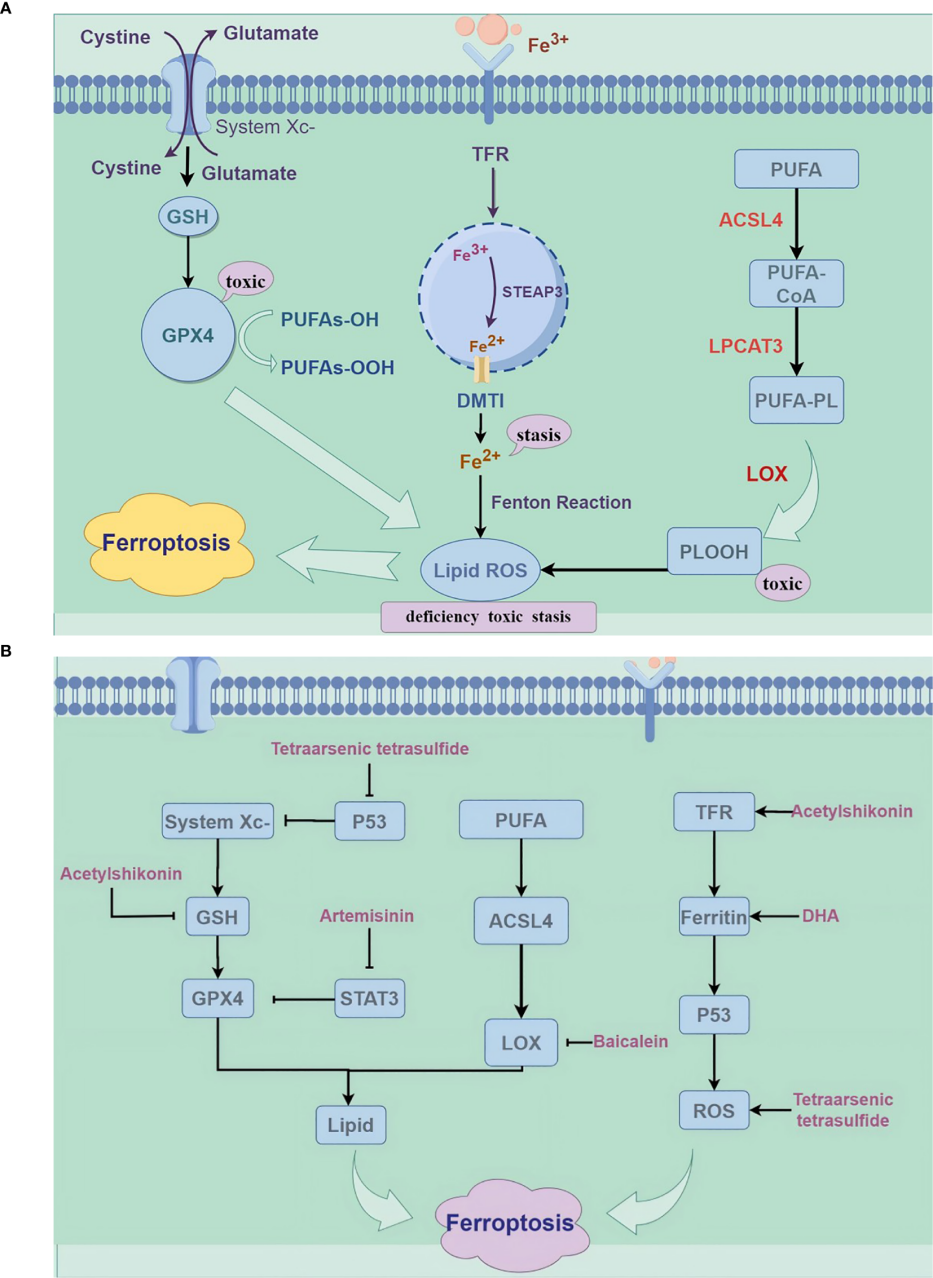
**(A)** Mechanisms of ferroptosis (by Figdraw). ROS, reactive oxygen species; GPX4, glutathione peroxidase 4; GSH, glutathione; PUFA, Polyunsaturated Fatty Acids; LPCAT3, lysophospholipid acyltransferase 3; ACSL4, long-chain esteroyl coenzyme A synthetase 4; TFR, Transferrin Receptor; LOX, lipoxygenase. **(B)** The mechanism of traditional Chinese medicine compounds in ferroptosis (by Figdraw); DHA, dihydroartemisinin; STAT3, Signal Transducer and Activator of Transcription 3.

Stockwe’s laboratory demonstrated that the inhibition of system Xc- and GPX4 induces ferroptosis (13). System Xc- mediates the exchange of glutamate and cysteine across lipid membranes, ultimately facilitating the synthesis of reduced GSH. Inhibition of system Xc- reduces GSH expression, which leads to ferroptosis. Erastin triggers ferroptosis by blocking the system Xc- pathway (14). GPX4 catalyzes the conversion of reduced GSH to oxidized GSH while reducing PL-OOH to PL-OH (12). An increase in GPX4 activity reduces phospholipid hydroperoxide levels, ultimately leading to ferroptosis (15). It was found that lipid peroxidation and the accumulation of intracellular reactive oxygen species can be promoted by knocking down the mouse membrane lipid repair enzyme GPX4 or by the direct application of a GPX4 inhibitor (RSL3) (4); thus, GPX4 may serve as a key regulator of ferroptosis occurrence (**Figure 1**).

## 4 Ferroptosis and hematologic malignancies

Patients with hematologic malignancies often encounter a paradoxical challenge: the therapeutic measures required to combat their disease, such as repeated blood transfusions during chemotherapy, inadvertently lead to increased iron levels. Unlike in solid tumors, this iron burden is not merely a bystander effect; it actively contributes to disease progression. The elevated iron levels enhance intracellular ROS, catalyzing the Fenton reaction and promoting ferroptosis (16). This iron-driven ferroptosis suggests a vicious cycle in which primary bone marrow dysfunction leads to iron overload, further exacerbating marrow pathology. However, the mechanisms regulating the development of hematological tumor cells through ferroptosis remain unclear. Therefore, it is essential to further investigate and explore the specific mechanisms of cellular ferroptosis to regulate hematological tumor cells and identify related markers for ferroptosis detection.

### 4.1 Ferroptosis and acute myeloid leukemia

Acute myeloid leukemia (AML) remains one of the most common hematologic malignancies, with iron overload identified as a significant risk factor for its development. In leukemia patients, excess iron contributes to immune system evasion and increases the probability of infection by inducing abnormalities in T-lymphocyte subpopulations and increasing the proportion of regulatory T cells (17, 18). This process is also accompanied by a significant increase in ROS, leading us to hypothesize that iron overload regulates T lymphocytes through enhanced ROS production. Innovative research by Yu et al. (19) has demonstrated that the targeting system xc- with the inhibitor Erastin induces ferroptosis in HL-60 cells *in vitro*, offering a potential new therapeutic avenue for leukemia. Similarly, eprenetapopt (APR-246), a small molecule capable of restoring the activity of mutant p53 and inducing apoptosis, acts independently of TP53 mutations and relies on intracellular GSH depletion and lipid peroxidation (20); thus, this treatment could be widely applicable among different patients. Rushidia et al. found that leukemia cells exhibit resistance to Aldh3a2 (aldehyde dehydrogenase 3a2), an enzyme that oxidizes long-chain aliphatic aldehydes to prevent oxidative cellular damage. The absence of Aldh3a2 leads to ferroptosis-dependent oxidative cell death in leukemia cells, while normal hematopoiesis remains unaffected. GPX4 inhibition, a known trigger of iron overload, has minimal effects on its own; however, when both Aldh3a2 and GPX4 are inhibited, a synergistic effect is evident, resulting in increased cell killing. Therefore, Aldh3a2 inhibition in combination with ferroptosis inducers may represent a promising approach for leukemia therapy (21).

### 4.2 Ferroptosis and acute lymphoblastic leukemia

Acute lymphoblastic leukemia (ALL) is the most prevalent malignancy in children, though it can also manifest in adults across all age groups. Liu et al. (22) treated Molt-4 cells, a human leukemia cell line, with erastin, leading to increased levels of reactive oxygen species and malondialdehyde. They found that erastin induces ferroptosis in Molt-4 cells, a process that can be partially reversed by the ferroptosis inhibitor ferrostatin-1 and the p38 MAPK inhibitor. Furthermore, Probst et al. (23) utilized ALL cells as a model and observed cell death following RSL3 treatment, which was accompanied by increased lipid peroxidation. The introduction of the lipid peroxidation inhibitor Fer-1 or lipoxygenase (LOX) effectively prevented cell death, while the iron chelator Deferoxamine (DFO) reversed RSL3-triggered cell death. These findings suggest that ALL cells exhibit sensitivity to RSL3-induced ferroptosis.

### 4.3 Ferroptosis and myelodysplastic syndromes

Myelodysplastic syndromes (MDS) are a group of myeloid clonal disorders that originate from hematopoietic stem cells. A significant complication associated with MDS is iron overload, which can disrupt the bone marrow microenvironment. This disruption leads to decreased viability of bone marrow mononuclear cells, a reduction in GSH and GPX4 activity, and an increase in ROS. In MDS, ROS can cause indiscriminate oxidative damage to DNA, lipids, and proteins, potentially increasing the risk of leukemic transformation in affected patients (24). Decitabine, a key therapeutic agent for high-risk MDS, has been shown to elevate ROS levels within the bone marrow cells of MDS patients by down-regulating GSH and GPX4 levels, which subsequently leads to ferroptosis. However, this process can be inhibited by agents such as ferroportin-1, necroptin-1, and Z-VAD-FMK (25).

### 4.4 Ferroptosis and multiple myeloma

Multiple myeloma (MM), a prevalent hematologic cancer affecting plasma cells within the bone and bone marrow, is the second most commonly diagnosed blood malignancy. Zhong et al. (26) found that fingolimod (FTY720), a novel immunosuppressant, can inhibit ROS clearance by decreasing the levels of GPX4 and SLC7A11. The accumulation of intracellular Fe2+ induced by FTY720 may enhance ROS production, ultimately leading to iron dysregulation in multiple myeloma cells. Zhang et al. (27) reported that ACSL4 is aberrantly overexpressed in MM cell lines and patients. ACSL4 serves as a promoter of ferroptosis, influencing the sensitivity of MM cells to RSL3, a known ferroptosis inducer. The knockdown of ACSL4 decreased the susceptibility of MM cells to ferroptosis, positioning ACSL4 as a potential therapeutic target in this disease. Furthermore, Li et al. (28) discovered that andrographolide (Andro) induces cell death, G0/G1 cell cycle arrest, and oxidative stress in MM cells through an increase in intracellular and mitochondrial iron and lipid peroxidation levels. Ferroptosis inhibitors may be effective in treating Andro-induced cell death, and it was also found that Andro induces ferroptosis in MM cells via the P38/Nrf2/HO-1 pathway.

## 5 Therapeutic role of Chinese medicine in intervening ferroptosis in hematologic malignancies

Traditional Chinese Medicine (TCM) has a rich historical foundation in the treatment of hematologic malignancies. The emergence of advanced molecular biology techniques has positioned TCM at the forefront of contemporary research, especially regarding the molecular modulation of hematological diseases. This review examines the implications of TCM in iron-related cell death by analyzing the TCM pathomechanism of blood disorders, with a focus on the role of TCM-derived monomers and bioactive compounds in the ferroptosis pathway.

### 5.1 Chinese medicine association of iron death and hematologic malignancies

Hematologic malignancies involve a wide range. The clinical symptoms are mostly bleeding, fever, fatigue, etc., so they can be classified as deficiency diseases (29). The essence is the abnormal operation of Qi and blood (30). The pathogenesis is mainly based on the deficiency of spleen and kidney, and the internal blockage of stasis and toxin, where deficiency is the basis, and “toxin and stasis” are the reality. Based on the analysis of ferroptosis theory of traditional Chinese medicine, it can be seen that the excessive iron and lipid peroxides in the body are deposited in the blood vessels, which hinders the normal operation of Qi and blood, blocks the blood circulation, leads to meridian obstruction, and further emphasizes the imbalance of Yin and Yang in the human body. With the further development of the disease, the pathological products accumulate in large quantities, and the lipid peroxidation reaction occurs under the catalysis of iron, producing toxic lipid peroxides, which accumulate into toxin. The toxin further invades the human body, the healthy gradually declines, further consumes Qi and blood, aggravates the symptoms of bleeding, infection and fever. There exists a notable correlation between MDS iron overload and blood stasis syndrome as understood in traditional Chinese medicine. Iron overload heightens the risk of infection in patients following transplantation. Consequently, it is crucial to examine the treatment of hematologic malignancies through the lens of traditional Chinese medicine, particularly in relation to ferroptosis. Furthermore, future research should delve deeper into the biological foundations of stasis and toxicity, thereby providing a framework for traditional Chinese medicine to effectively regulate ferroptosis in hematologic malignancies.

### 5.2 Chinese medicine active ingredients

#### 5.2.1 Artemisinin

The extracts and principal components of Artemisia annua exhibit a range of biological activities, including anti-malarial, antitumor, bacteriostatic, insecticidal, antipyretic, anti-inflammatory, and immunomodulatory effects (48–51). Research indicates that artemisinin and its derivatives can induce ferroptosis in tumor cells, acting as iron death inducers (31). In a study by Lai et al. (32), it was demonstrated that artemisinin, when combined with transferrin, can effectively and selectively target human leukemia Molt-4 tumor cells, exhibiting minimal toxicity towards normal cells. Furthermore, artemisinin analogs (33) are primarily utilized in the treatment of leukemia, lymphoma, and multiple myeloma by inducing cell cycle arrest in the G0/G1 phase, inhibiting proliferation and angiogenesis, and down-regulating VEGF and associated signaling pathways. Lu et al. (34) found that dihydroartemisinin (DHA) induces apoptosis in HL-60 leukemia cells, characterized by a decrease in mitochondrial membrane potential and the activation of both Caspase-9 and Caspase-8. Additionally, DHA can trigger cellular ferroptosis by modulating the mTOR/p70S6k signaling pathway and activating the autophagic degradation of ferritin heavy chain (FTH) in AML cells. Both DFO and Fer-1 have been shown to mitigate DHA-induced cell death (35).

#### 5.2.2 Baicalein

Baicalein and baicalin are recognized for their diverse pharmacological effects, which include antibacterial, antiviral, antitumor, anti-inflammatory, and antioxidant properties. Additionally, they are known to protect liver function, dissipate heat, relieve pain, and are utilized in the treatment of cardiovascular diseases. Zhao Yuling et al. (36) concluded that baicalin can scavenge free radicals and reactive oxygen species, demonstrating a protective effect against oxidative damage induced by iron overload. Li Ming et al. (37) showed that baicalein increases the expression of GPX4 and xCT, with GPX4 utilizing GSH as a reducing agent to convert lipid peroxidation products into alcohols, thereby protecting cells from ferroptosis. Furthermore, baicalein (38) can directly chelate iron ions in human tissues, reducing the deposition of these ions in the body and thereby safeguarding the organism from the damage associated with iron ion accumulation. Yu et al. found that baicalein exhibits significant anti-AML activity both *in vitro* and *in vivo *(39), inhibiting AMLK cell proliferation by inducing cell cycle arrest and differentiation, thus establishing it as an effective therapeutic agent for the treatment of AML.

#### 5.2.3 Acetylshikonin

Acetylshikonin, a naphthoquinone compound derived from the traditional Chinese herb comfrey, has been recognized for its significant roles in anticancer (40) and antiviral (41) research. One study demonstrated (42) that acetylshikonin induced apoptosis in K562 cells, which was accompanied by a rapid generation of ROS. Additionally, acetyl viologen significantly induced apoptosis by regulating ROS accumulation, depleting Bcr-Abl, and blocking the NF-κB signaling pathway, indicating its therapeutic potential for treating chronic myeloid leukemia (CML) patients. Li et al. (43) found that acetyl viologen could promote ferroptosis in human leukemia HL-60 cells by decreasing intracellular GSH levels and inhibiting the expression of GPX4, with these changes being reversible through the application of ferroptosis inhibitors. Furthermore, acetylshikonin has been established as an active ingredient in traditional Chinese medicine, demonstrating the capability to inhibit the proliferation of HL-60 cells in acute promyelocytic leukemia.

#### 5.2.4 Arsenic

Arsenic trioxide, denoted as As2O3, is a compound recognized for its significant toxicity. Various types of ROS can be generated during arsenic metabolism in cells (44). GSH is utilized as an electron donor, or arsenic may bind to its sulfhydryl group. Both chronic exposure to arsenic and acute exposure at high doses can provoke GSH depletion; elevated ROS levels, coupled with reduced GSH, can instigate lipid peroxidation. Bai et al. (45) observed an induction in the expression of GSH-related proteins following arsenic exposure, noting that arsenic metabolism generates an overproduction of ROS, thereby disrupting antioxidant homeostasis, activating apoptosis-related cysteine proteases, and inducing apoptotic processes. Lipid peroxidation is an essential mechanism of ferroptosis. The precise mechanism of arsenic-induced apoptosis remains to be elucidated. It is plausible to conjecture that arsenic may elicit ferroptosis in hematological tumor cells, a hypothesis that warrants further investigation. Realgar, initially documented in the Shen Nong Ben Cao Jing (Classic of the Materia Medica of the Divine Husbandman), predominantly comprises tetrasodium arsenic tetrasulfide (As4S4). It has been shown that microbial extracts of Realgar can effectively inhibit the proliferation of HL-60 cells, regulate the cell cycle, and induce apoptosis (46). Tetraarsenic tetrasulfide can cause ferroptosis by activating ROS/p53 axis in B-ALL cell lines (47). Qinghuang Powder, which includes Realgar as a principal component, has been widely used in treating leukemia and MDS. The potential involvement of ferroptosis in its therapeutic action invites further exploration (**Table 1**).

**Table 1:** Molecular mechanism of traditional Chinese medicine active ingredients for treatment of hematology diseases by intervention in ferroptosis pathway.

Traditional Chinese medicine monomer	Chinese Medicine Active Ingredients	Research model	Action mechanism	Reference
Sweet wormwood	Artemisinin	lymphoma cell lines DAUDI and CA-46 cells of mouse	activate the ATF4-CHOP-CHAC1 pathway then enhanced ferroptosis	(48)
		lymphoma cell lines DLBCL	induces ferroptosis by impairing STAT3 signalin	(49, 50)
	Dihydroartemisinin	human ALL cell lines (HL-60, THP-1, KG1)	enhance ferritin degradation, labile iron pool, and ROS accumulation	(35, 51)
Skullcap	Baicalein	RSL3-stimulated HT22 cells of mouse	increase the expression of GPX4 and xCT	(37)
		human T-cell ALL cell lines Jurkat and Molt-4	reduce lipid peroxidation and ROS accumulation	(23)
Alkanet	Acetylshikonin	human leukemia HL-60 cells	inhibiting the expression of GPX4 down-regulating the expression of FTH1	(43)
Realgar	Tetraarsenic tetrasulfide	human B-ALL cell lines	through the ROS/p53 signaling pathway	(47)

ROS, reactive oxygen species; GPX4, glutathione peroxidase 4; xCT, System Xc-; GSH, glutathione; DCBCL, Diffuse Large B-Cell Lymphoma; FIH1, ferritin heavy chain 1.

## 6 Discussion

Ferroptosis, a form of cell death characterized by lipid peroxidation and increased iron dependency, has emerged as a focal point of contemporary research. The pathogenesis of ferroptosis is complex, involving both independent and interrelated mechanisms that form a sophisticated regulatory network. Numerous studies have confirmed that ferroptosis plays a crucial role in the development of hematologic malignancies, and that intervening in the ferroptotic process can mitigate the progression of related diseases to varying extents. Novel ferroptosis inhibitors and activators have become a significant area of interest in cellular and chemical biology, offering new strategies for the targeted therapy of hematologic malignancies. However, our understanding of iron metabolism disorders and ferroptosis in hematologic conditions remains preliminary, highlighting the need for further investigation. Traditional Chinese Medicine (TCM) is an emerging option for treating hematologic malignancies. The active ingredients of TCM, such as artemisinin, baicalin, acetylviologen, and arsenic, have been widely utilized in clinical practice. Nevertheless, existing studies on the pathways through which TCM regulates ferroptosis primarily focus on individual monomers or active ingredients. More comprehensive research is required on both basic and clinical interventions involving TCM formulations and acupuncture in relation to ferroptosis. Most of the current studies are experimental, and evidence from clinical studies is relatively scarce. Therefore, the development of relevant clinical studies is a crucial direction for future research. Nonetheless, the current research on ferroptosis is still in its infancy, necessitating advancements in identifying relevant mechanistic targets. In summary, regulating ferroptosis holds significant potential for the treatment of hematologic malignancies and offers a new avenue for targeted therapies within TCM.
